# Silvopastoral system is an alternative to improve animal welfare and productive performance in meat production systems

**DOI:** 10.1038/s41598-021-93609-7

**Published:** 2021-07-08

**Authors:** Amanda Prudêncio Lemes, Alexandre Rossetto Garcia, José Ricardo Macedo Pezzopane, Felipe Zandonadi Brandão, Yeda Fumie Watanabe, Reinaldo Fernandes Cooke, Mariana Sponchiado, Claudia Cristina Paro de Paz, Annelise Carla Camplesi, Mario Binelli, Lindsay Unno Gimenes

**Affiliations:** 1 Faculdade de Ciências Agrárias e Veterinárias, Universidade Estadual de São Paulo “Júlio de Mesquita Filho”, Rod. Prof. Paulo Donato Castellane, s/n, Jaboticabal, SP 14884-900 Brazil; 2 Embrapa Pecuária Sudeste, Rod. Washington Luiz, km 234, Fazenda Canchim, PO Box: 339, São Carlos, SP 13560-970 Brazil; 3 Universidade Federal Fluminense, Rua Vital Brazil, 64, Niterói, RJ 24230-340 Brazil; 4 Vitrogen, Av. Coronel José Nogueira Terra, 203, Cravinhos, SP 14140-000 Brazil; 5 Texas A&M University, 400 Bizzell St, College Station, TX 77843 USA; 6 University of Florida, PO Box 110910, Gainesville, FL 32611 USA; 7 Instituto de Zootecnia, Rod. Carlos Tonani, km 94, Sertãozinho, SP Brazil

**Keywords:** Climate-change mitigation, Animal biotechnology, Metabolism, Reproductive biology, Animal physiology

## Abstract

Climate change is a reality and global surface temperature is projected to rise substantially in the next 80 years. Agriculture practices will have to adapt to climate change, and also help to mitigate this effect using, among other strategies, forest conservation and management. Silvopastoral systems have been adopted in tropical climate livestock areas but their benefits on thermal comfort and reproductive performance of beef cows are not completely known. Therefore, our aims were to compare the microclimate of silvopastoral and intensive rotational unshaded grazing systems in different months and to evaluate physiological variables (Exp. 1 and 2), metabolism, and in vitro embryo production (Exp. 2) in crossbred beef females. Our hypothesis is that the silvopastoral system can improve the thermal comfort of beef heifers and cows and, consequently, also improve dry matter intake, body weight gain, and in vitro embryo production when compared to the unshaded rotational grazing system. In Exp 1, the silvopastoral system decreased body temperature and increased welfare and performance of heifers. In Exp. 2, the silvopastoral system enhanced the body weight but did not affect metabolism and the general reproductive performance, but increased the recovery rate of oocytes in primiparous cows.

## Introduction

It is expected that in 2050 the world population will reach more than 9 billion people. Urbanization will continue to grow at an accelerated rate and the increase in income per individual will change dietary requirements and preferences to include more diverse food sources of greater nutritional value^1^. Furthermore, the growing urban population has an increased concern about the sources of the food supply and a greater awareness of animal welfare. In order to meet this upcoming challenge and the modified consumer perceptions, it will be necessary to increase food production by 70%^2^. To that end, modern production strategies that combine sustainability, as well as technical and economic viability, have the potential to increase livestock productivity in the face of climate change and are pivotal to the environment and to meet the future demands for food.

For decades, meat and dairy production in the tropics has been based on the binomial animal performance and individual thermotolerance. However, bovine is reported to have a genetic antagonism between heat stress tolerance and high milk or meat production. Thus, scientific and technological advances have attempted to modify the environment for the animal, in order to ensure a greater expression of the genetic potential and, consequently, increased production^3^.

Grassland systems are the base of dairy and beef production in the tropics^4^. In recent years, systems that integrate crop, livestock, and forest have gained prominence as production models in the tropics^5^. Among them are the silvopastoral systems, which are agroforestry arrangements that purposely combine fodder plants, such as grasses and leguminous herbs, with shrubs or trees for animal feeding and complementary uses^6,7^. Some benefits in the production of cattle managed in areas that integrate grasses and arboreal components have been reported^7^, emphasizing the role of shade provided by trees as an efficient component that increases comfort and animal welfare^8^.

Under thermal comfort, both dairy^9^ and beef cattle^10^ achieve greater productivity. Furthermore, serum concentrations of cortisol are lower in animals under mild temperatures when compared to heat-stressed animals^11^. On the other hand, metabolic changes due to increased body temperature can reduce the reproductive efficiency of heat-stressed animals^12,13^. Thermal stress also has negative effects on oocyte quality, resulting in lower rates of in vitro embryo production in Bos taurus^12,14,15^.

**Figure 1:**
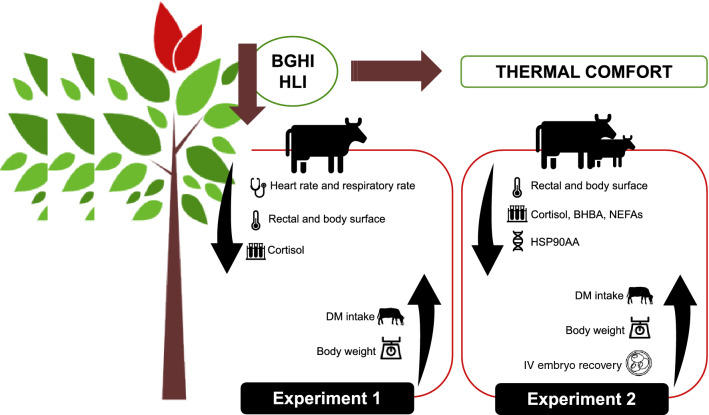
Hypothetical model. Natural shade in pasture areas improves the thermal comfort of beef heifers and beef cows due to both reduced black globe humidity index (BGHI) and heat load index (HLI). It consequently determines reduction in heart rate, respiratory rate, rectal and body surface temperatures, cortisol, heat shock proteins expression (HSP90AA) and increases dry matter intake (DMI), body weight, and in vitro embryo recovery. *BHBA* β-hydroxybutyrate, *NEFAs* non-esterified fatty acids.

In spite of the increasing adoption of silvopastoral systems in the tropics^4^, scientific reports about the benefits that integrate natural shading may offer to beef heifers and cows are scarce in the literature. Moreover, there is a lack of information, especially concerning physiological variables related to female thermal balance and their reproductive variables, such as oocyte quality and embryo production. Therefore, the objectives of this study were to compare silvopastoral to conventional unshaded grazing beef production systems considering: (a) the thermal comfort of beef heifers and primiparous cows; and (b) the metabolic profile, oocyte quality, and in vitro embryo production of beef cows. The basic hypothesis is that the silvopastoral system can improve the thermal comfort of beef heifers and cows and, consequently, also improve dry matter intake, body weight gain, and in vitro embryo production when compared to the unshaded rotational grazing system. In order to test the validity of this hypothesis, two sequential and complementary experiments were designed, by evaluating the thermal comfort (Experiment 1), and the metabolic and reproductive responses (Experiment 2) of dams raised on silvopastoral versus intensive unshaded rotational systems over time (Fig. 1).

## Results

### Experiment 1

#### Microclimate

**Figure 2:**
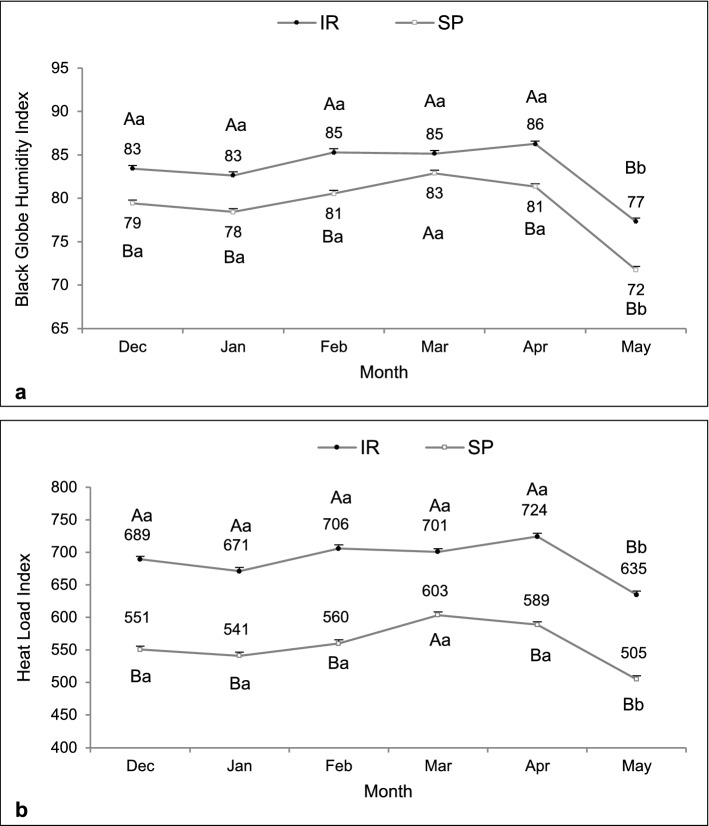
Microclimate characterization of pasture systems in silvopastoral (SP) or intensive rotational (IR) grazing systems according to black globe humidity index (**a**) and heat load index (**b**) registered from December 2015 to May 2016. There was an interaction between system and month on black globe humidity index and heat load index. ^A,B^Different capital letters indicate a significant difference between systems. ^a,b^Different lowercase letters indicate a significant difference between months.

The average solar radiation transmission in the silvopastoral system (SP) was 37.6% in comparison to solar radiation transmission of 100% in the intensive rotational system (IR). Therefore, the forested system had a shading efficiency of 62.4%. There was an interaction between system and month on black globe humidity index (F = 5.36, df = 660, P < 0.01) and heat load index (F = 9.74, df = 660, P < 0.01). The SP system had both indexes lower than the intensive rotational system along all months, except in March when an increase was observed. In May, a decrease was observed in the IR system (Fig. 2a,b).

#### Physiological variables

**Figure 3:**
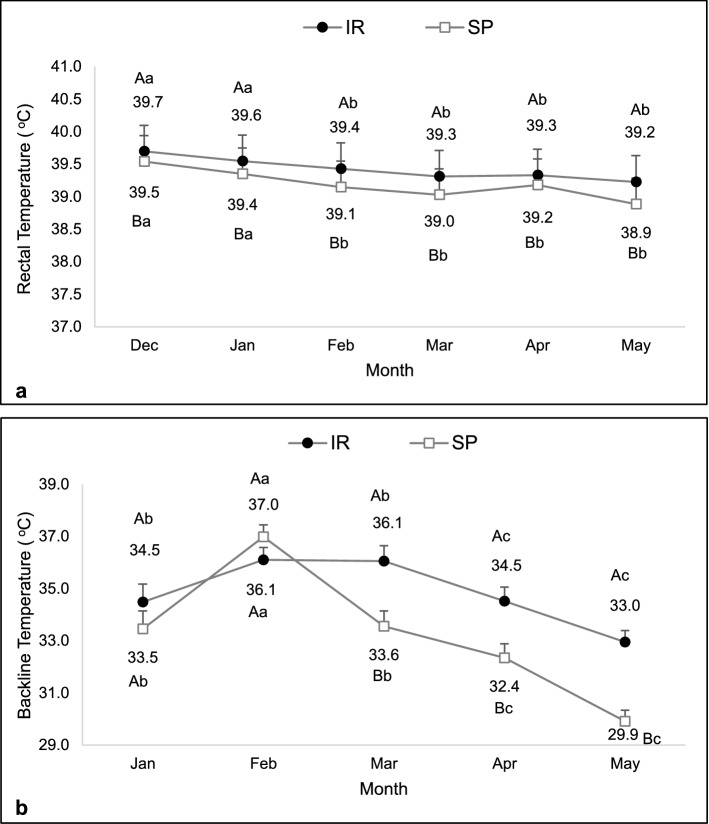
Rectal (**a**) and back line (**b**) temperatures of heifers kept in the silvopastoral (SP) or intensive rotational (IR) grazing systems. For rectal temperature, main effects were observed. For backline temperature, an interaction between system and month was observed. ^A,B^Different capital letters indicate a significant difference between systems. ^a,b,c^Different lowercase letters indicate a significant difference between months.

No interaction between system and month was observed for rectal temperature (F = 0,62, df = 179, P = 0.69), which was higher in heifers in the IR than in the SP system (39.4 ± 0.07 vs 39.2 ± 0.07 °C; F = 34.09, df = 181, P < 0.01). Regarding months, the rectal temperature was higher in December and January (F = 16.83, df = 179, P < 0.01; Fig. 3a). There was an interaction between system and month for backline temperature (F = 4.09, df = 96, P < 0.01; Fig. 3b). This temperature was similar between systems in January and February and then decreased in heifers raised in the SP system in March, April, and May.

Respiratory rate and heart rate were not influenced by the systems nor the system and month interaction. In January and February, the heart rate was higher; however, the respiratory rate remained similar in all months of the study (Table 1).

**Table 1:** Least square mean ± standard error of the physiological responses of heifers in silvopastoral (SP; n = 16) or intensive rotational grazing (IR; n = 16) systems.

Factors	Variables^c^	
	RR (breaths/min)	HR (beats/min)
**System**		
SP	46.4 ± 1.0	96.5 ± 1.2
IR	44.4 ± 1.0	93.1 ± 1.2
**Month**		
December	45.4 ± 1.7	83.0 ± 2.1^b^
January	48.3 ± 1.9	100.9 ± 2.2ª
February	44.2 ± 1.9	105.2 ± 2.2ª
March	44.6 ± 1.7	95.0 ± 2.1^b^
April	43.8 ± 1.7	94.4 ± 2.1^b^
May	46.1 ± 1.9	90.3 ± 2.2^b^
**P values**^d^		
S^e^	0.19	0.06
M^f^	0.52	< 0.01
S*M^g^	0.45	0.61

^a,b^Means followed by different lowercase letters on the same column are significant.

^c^*RR* respiratory rate, *HR* heart rate, ^d^P values < 0.05 were considered significant. ^e^*S* system, ^f^*M* month (experimental month), ^g^S*M interaction between system and month.

**Figure 4:**
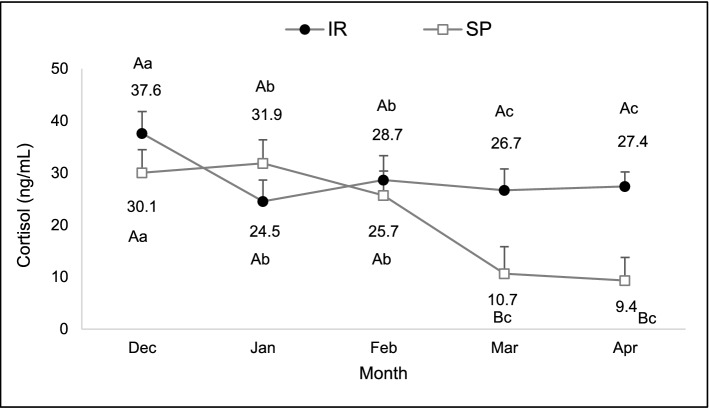
Serum cortisol concentration of heifers raised on silvopastoral (SP) or intensive rotational (IR) systems from December to April. There was an interaction between system and month. ^A,B^Different capital letters indicate a significant difference between systems. ^a,b,c^Different lowercase letters indicate a significant difference between months.

There was an interaction between system and month in cortisol levels (F: 2.84, df: 123, P = 0.03). Heifers of the SP system presented lower concentrations in March and April than those of the IR (Fig. 4).

#### Productive performance

**Figure 5:**
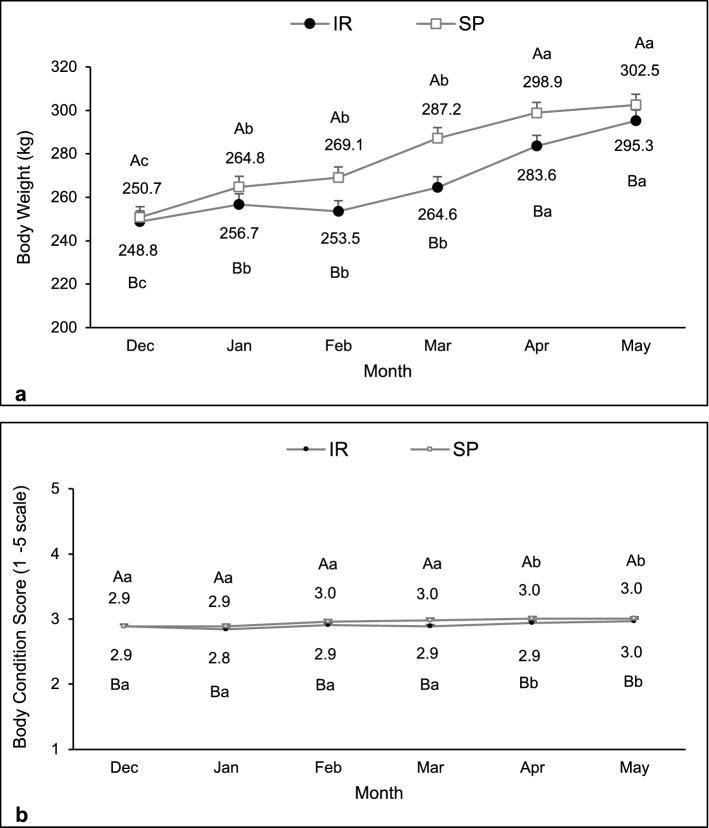
Body weight (**a**) and body condition score (**b**) evolution of heifers kept in the silvopastoral (SP) or intensive rotational (IR) grazing systems from December 2015 to May 2016. There was a significant effect of system and month on body weight and body condition score. ^A,B^Different capital letters indicate a significant difference between systems. ^a,b,c^Different lowercase letters indicate a significant difference between months.

No interactions between system and month were observed for body weight (F = 1.16, df = 180, P = 0.33), body condition score (F = 0.70, df = 185, P = 0.63), and dry matter intake (F = 1.10, df = 180, P = 0.36). There was a significant effect of system on body weight (F = 17.55, df = 180, P < 0.01) and body condition score (F = 9.12, df = 186, P < 0.01), in which in the SP it was higher than in the IR system. Besides that, these variables were similar at the beginning of the experiment and gradually increased in April and May in both systems. (BCS: F = 5.34, df = 185, P < 0.01; BW: F = 30.83, df = 180, P < 0.01; Fig. 5a,b).

Dry matter intake was higher in the SP than in the IR system (6.5 ± 0.03 vs 6.4 ± 0.03; F = 17.67, df = 180, P < 0.01). Regardless of system, dry matter intake was higher in March (6.4^b^ ± 0.06), April (6.8^a^ ± 0.06), and May (6.7^a^ ± 0.06) than in December (6.1^c^ ± 0.06), January (6.2^c^ ± 0.06), and February (6.2^c^ ± 0.06); F = 22.84, df = 180, P < 0.01).

### Experiment 2

#### Microclimate

**Figure 6:**
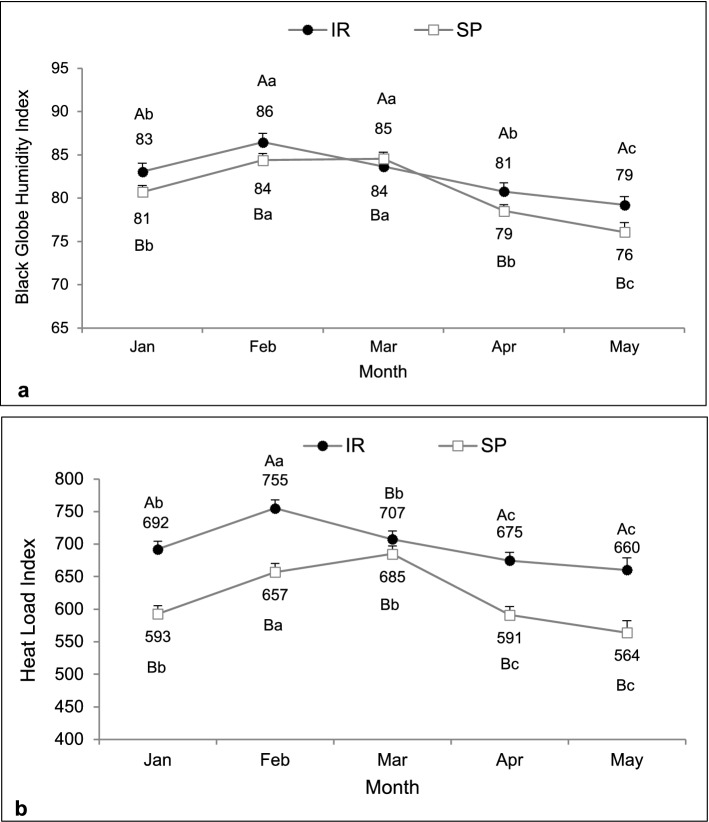
Microclimatic characterization of pasture systems in silvopastoral (SP) or intensive rotational (IR) grazing systems according to black globe humidity index (**a**) and heat load index (**b**) recorded from January to May 2017. Main effects were observed for black globe humidity index, while an interaction was observed for heat load index. ^A,B^Different capital letters indicate a significant difference between systems. ^a,b,c^Different lowercase letters indicate a significant difference between months.

The solar radiation in the SP was 56.9% in comparison to the solar radiation transmission of 100% in the IR system. Therefore, the system had a shading efficiency of 43.1%. Black globe humidity index was higher in the IR than in the SP system (F = 11.67, df = 257, P < 0.01). Regardless of the systems, this index was higher in February and March (F = 24.55, df = 257, P < 0.01; Fig. 6a). There was an interaction between system and month for heat load index, which was higher in the IR, except for March, when it was similar to the SP system (F = 3.39, df = 258, P = 0.01; Fig. 6b).

#### Physiological variables

**Figure 7:**
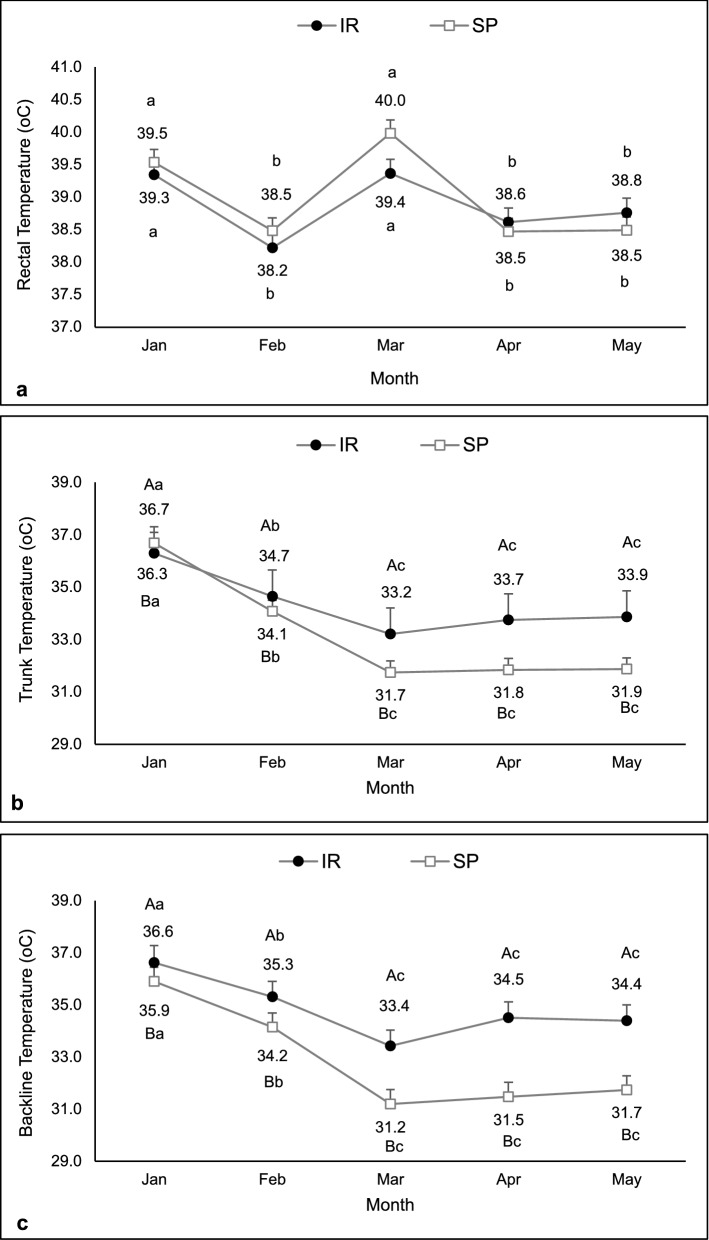
Rectal (**a**), trunk (**b**), and back line (**c**) temperatures of cows kept in the silvopastoral (SP) or intensive rotational (IR) grazing systems from January to May 2017. An effect of month was observed for rectal temperature. Trunk and back line temperatures were affected by both main effects. ^A,B^Different capital letters indicate a significant difference between systems. ^a,b,c^Different lowercase letters indicate a significant difference between months.

Rectal temperature was affected only by month and not by system nor by system and month interaction (F = 9.02, df = 79, P < 0.01, F = 0.28, df = 79, P = 0.60, and F = 0.87, df = 79, P = 0.49, respectively). A higher internal temperature was observed in January and March (Fig. 7a). The SP system determined lower trunk (F = 21.18, df = 77, P < 0.01) and back line (F = 28.39, df = 77, P < 0.01) temperatures during the experimental period. Higher trunk (F = 21.13, df = 77, P < 0.01) and back line (F = 14.84, df = 77, P < 0.01) temperatures were observed in January and February (Fig. 7b,c).

#### Cortisol and heat shock proteins

**Figure 8:**
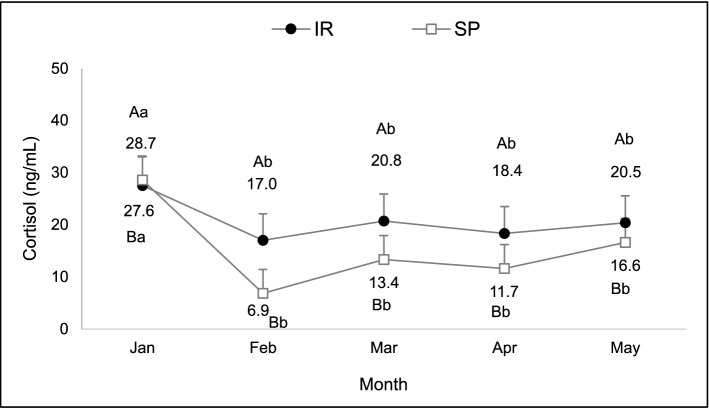
Serum cortisol concentration of heifers kept in the silvopastoral (SP) or intensive rotational (IR) grazing systems from January to May 2017. Cortisol levels were influenced by the system (P = 0.08) and the month (P = 0.02). ^A,B^Different capital letters indicate a significant difference between systems. ^a,b,c^Different lowercase letters indicate a significant difference between months.

No interaction was observed for cortisol (F = 0.37, df = 79, P = 0.83). There was a tendency of increased cortisol levels for cows raised in the IR compared to the SP system (F = 3.05, df = 79, P = 0.08) and a higher mean serum cortisol level was observed in January (F = 2.99, df = 79, P = 0.02; Fig. 8). Abundance of HSP90AA1 was not different between SP and IR systems, nor months or interaction (F = 0.73, df = 74, P = 0.40; F = 0.83, df = 74, P = 0.51; and F = 0.60, df = 74, P = 0.66, respectively).

#### Productive performance

**Figure 9:**
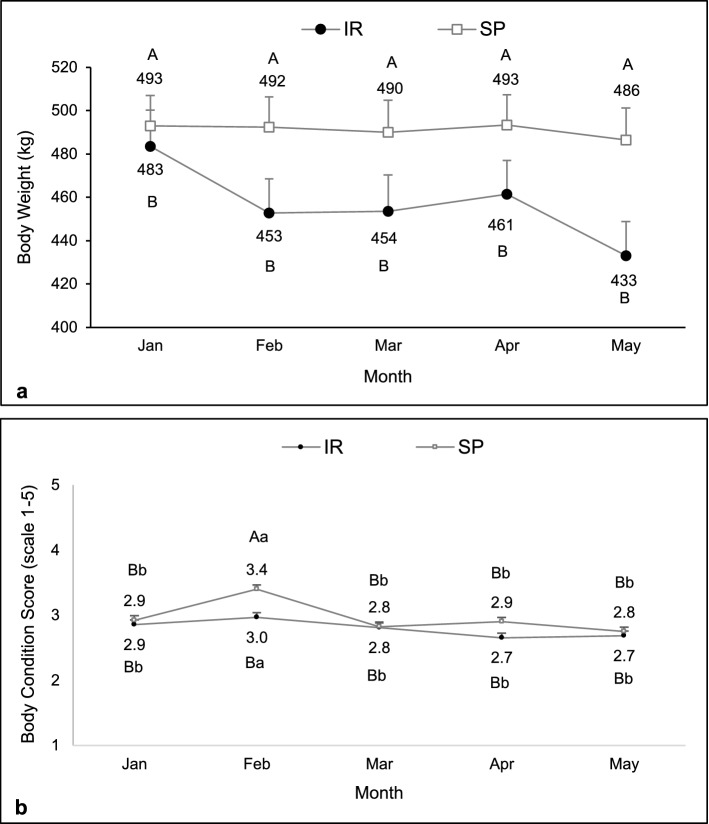
Body weight (**a**) and body condition score (**b**) of females kept in the silvopastoral (SP) or intensive rotational grazing (IR) systems from January to May 2017. Body weight was influenced by system, and body condition score by an interaction between system and month. ^A,B^Different capital letters indicate a significant difference between systems. ^a,b,c^Different lowercase letters indicate a significant difference between months.

Body weight was higher in cows maintained in silvopastoral than intensive rotational system (F = 12.53, df = 76, P < 0.01; Fig. 9a); however, no effect of month or interaction was observed (F = 0.90, df = 76, P = 0.47 and F = 0.54, df = 76, P = 0.71, respectively). An interaction between system and month was observed for body condition score (F = 3.08, df = 79, P = 0.02), in which cows in the silvopastoral showed similar scores than those in the intensive rotational system, except in February when they were higher (Fig. 9b).

Dry matter intake was not different between SP and IR systems (9.0 ± 0.08 vs 8.9 ± 0.10 kg; F = 0.82, df = 76, P = 0.37) nor among experimental months (F = 1.51, df = 76, P = 0.21). Calves of cows kept in SP or IR systems had similar body weight. However, male calves had heavier birth and weaning weights than female calves (Table 2).

**Table 2:** Least square mean ± standard error of the calf’s weight at birth and weaning according to gender and system (SP: silvopastoral; IR: intensive rotational grazing).

Factors	Variables^c^	
	Birth W (kg)	Weaning W (kg)
**System**		
SP	36.6 ± 1.8	176.4 ± 7.9
IR	38.4 ± 2.0	175.1 ± 8.6
**Gender**		
Female	33.6 ± 1.9^b^	161.5 ± 7.9^b^
Male	41.3 ± 2.7^a^	190.0 ± 8.6^a^
**P values**^d^		
S^e^	0.50	0.91
G^f^	0.01	0.03
S*G^g^	0.65	0.52

^a,b^ = means followed by different lowercase letters on the same column are significant.

^c^*Birth W* weight at birth, *Weaning W *weight at weaning, ^d^P values < 0.05 were considered significant; ^e^*S* system, ^f^*G *gender, ^g^S*G interaction between system and gender.

#### Metabolites and progesterone

**Figure 10:**
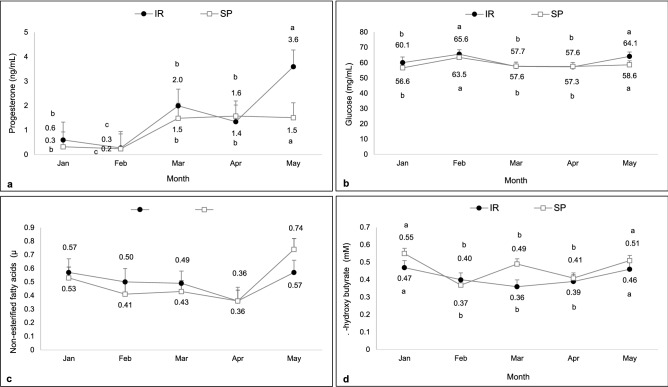
Serum concentration of progesterone (**a**), glucose (**b**), non-esterified fatty acids (**c**), and β-hydroxybutyrate (**d**) of beef cows kept in the silvopastoral (SP) or intensive rotational (IR) grazing systems from January to May 2017. All variables were affected by month. ^a,b,c^Different lowercase letters indicate a significant difference between months.

Serum levels of P4, glucose, BHBA, and NEFAs did not differ between SP and IR systems (F = 0.45, df = 79, P = 0.32, F = 0.98, df = 75, P = 0.51, F = 9.16, df = 79, P = 0.22, F = 0.12, df = 76, P = 0.95, respectively), and neither were influenced by the system and month interaction (F = 1.39, df = 79, P = 0.34; F = 0.42, df = 75, P = 0.45; F = 1.53, df = 79, P = 0.08; F = 0.58, df = 76, P = 0.56, respectively). Concentrations of P4 remained below 1 ng/mL in January and February. However, in March and April they increased and reached the highest concentration in May (F = 2.38, df = 79, P = 0.01). Glucose levels were higher in February and May (F = 2.86, df = 75, P < 0.01). NEFAs were lower in April (F = 2.99, df = 76, P = 0.01). Higher concentrations of BHBA were observed in January and May (F = 4.66, df = 79, P < 0.01; Fig. 10a–d).

#### Reproductive performance

Regarding ovum pick-up and in vitro embryo production, no interactions between system and month nor the effect of the system were observed for any variables analyzed (Table 3). The number of follicles observed was similar between months, however, total oocytes and viable oocytes were higher in January, March, and May. Recovery rate, cleaved structures, and cleavage rate were higher in March and May. The number of blastocysts and blastocyst rate were higher in May. The recovery rate of oocytes was higher in SP than in IR systems (74.3 ± 2.7 vs 65.3 ± 3.1%; F = 4.77, df = 79, P = 0.03).

**Table 3:** Least square means ± standard error of ovum pick-up-related variables and in vitro embryo production from primiparous Canchim cows kept in silvopastoral (SP) or intensive rotational grazing systems (IR).

Factors	Variables^d^							
	OF (n)	TO (n)	VO (n)	RC (%)	CS (n)	CR (%)	BD7 (n)	BR (%)
**System**								
SP	25.8 ± 2.1	20.9 ± 1.7	13.1 ± 1.3	74.3 ± 2.7^a^	11.8 ± 1.1	85.7 ± 2.6	5.1 ± 0.6	42.9 ± 4.0
IR	28.8 ± 2.4	20.2 ± 2.0	12.1 ± 1.4	65.3 ± 3.1 ^b^	10.3 ± 1.3	82.0 ± 3.0	4.1 ± 0.6	35.9 ± 4.6
**Month**								
January	30.5 ± 3.7	19.1 ± 3.0^ab^	13.0 ± 2.2^ab^	62.6 ± 4.6^bc^	11.0 ± 2.0^ab^	78.5 ± 4.7^bc^	3.9 ± 1.0^b^	34.8 ± 7.1^b^
February	20.1 ± 3.5	14.4 ± 2.9^b^	9.4 ± 2.1^ab^	64.3 ± 4.6^bc^	8.6 ± 1.9^b^	78.8 ± 4.6^bc^	3.1 ± 0.9^b^	31.3 ± 7.0^b^
March	28.5 ± 3.5	24.9 ± 2.9^ab^	16.9 ± 2.1^a^	79.0 ± 4.6^ab^	15.5 ± 1.8ª	92.7 ± 4.3^ab^	4.7 ± 0.9^b^	34.1 ± 6.5^b^
April	28.5 ± 3.5	16.6 ± 2.9^b^	8.5 ± 2.2^b^	57.3 ± 4.6^c^	6.1 ± 1.9^b^	75.3 ± 4.4^c^	2.6 ± 0.9^b^	38.6 ± 6.7^b^
May	28.8 ± 3.5	27.5 ± 2.9^a^	15.1 ± 2.1^ab^	85.7 ± 4.6^a^	14.1 ± 1.8^a^	94.1 ± 4.3^a^	8.6 ± 0.9^a^	58.3 ± 6.5^a^
**P value**^e^								
S^f^	0.35	0.79	0.61	0.03	0.35	0.36	0.24	0.25
M^g^	0.26	< 0.01	0.03	< 0.01	< 0.01	< 0.01	< 0.01	0.04
S*M^h^	0.87	0.99	0.86	0.32	0.84	0.22	0.94	0.67

^a,b,c^ = means followed by different lowercase letters on the same column are significant.

^d^*OF* observed follicles, *TO* total oocytes, *VO* viable oocytes, *RC* recovery rate of oocytes, *CS* number of structures cleaved, *CR* cleavage rate, *BD7* number of blastocysts in D7, *BR* blastocyst rate in D7, ^e^P values < 0.05 were considered significant, ^f^*S* system, ^g^*M* month (experimental month), ^h^S*M interaction between system and month.

## Discussion

Population growth and increased global demand for food require greater efficiency in production. Concurrently, it is necessary to increase the potential of production in the already anthropized areas, instead of exploiting untouched areas^1^. Benefits of using integrated production systems are often recorded in agronomic studies^6^; however, their direct effects on thermal stress mitigation, productive and reproductive performance of bovine females are not elucidated. In the present study, contemporary pasture systems with or without natural shade were compared, demonstrating that SP areas can improve the microclimate, determine welfare, and increase the productive performance of beef females.

The SP system mitigated the thermal challenge to the animals. This was confirmed by lower values of biometeorological indexes that are related to animal thermal comfort. Data corroborate similar studies in which significant reductions in temperature-humidity index^16–18^, black globe humidity index^18–20^, and heat load index^18,20,21^ were observed in forested pastures. There is a consensus that a black globe humidity index higher than 82 is considered harmful to productivity and requires additional mechanisms to animal thermoregulation^22^. Thus, the reduction promoted by the SP system provided a more comfortable microclimate^18,23^. As expected, in May, a transitional time between late autumn and early winter in the Southern hemisphere, both systems recorded the lowest black globe humidity index and heat load index values^24^. This fact is directly related to the natural reduction of solar radiation and air temperature, providing a milder climate^25^.

However, it is important to consider that during some specific periods of the year, depending on the tree arrangement, the apparent position of the sun can affect the level of radiation directly incident, influencing the comfort indexes. In Experiment 2, shading effectiveness in the SP system was 19.3% lower than in Experiment 1, due to the lower tree density adopted, justified by silvicultural management to maintain the development of plant components of the system. The increase in mean solar radiation within SP systems, although positive for pasture management, may reduce its efficiency to provide thermal comfort^20,24^ and, although this effect had not been observed in our study, it is necessary to consider this condition.

SP systems benefit animal thermal comfort^23,26^. Animal thermoregulation, which also comprises the loss of metabolic heat, depends on the flow of the body's heat to the environment^27^. In general, heifers and cows grazing in the SP had a lower internal and/or surface temperature than those grazing in the unshaded system. This indicates that, due to the better ambience provided by the SP system^20,21,28^, body heat loss was more efficient, corroborating previous studies in buffaloes^23^ and zebu heifers^29^. The response of body surface temperature to climatic variables is more instantaneous than core temperature variations^30,31^. This means that lower environmental temperatures result in lower surface temperature and favors thermal energy flow from the corporeal nucleus to the periphery, facilitating the process of conductive thermal changes^24,27^.

Nevertheless, considering the metabolic condition of lactating cows, rectal temperature may have been more susceptible to intrinsic factors related to the increase of the heat produced by the metabolism^9,14,15,23^. In this sense, it is admissible that the surface temperature is more sensitive than the internal temperature in terms of instantaneous microclimate changes. The SP system did not reduce respiratory and heart rate in heifers contrasting results in dairy cows^22,24^. However, mean heart and respiratory rates observed in heifers in both systems were higher than the range considered physiological (70 beats/min and 35 breaths/min, respectively)^32^. Heart rate is a physiological response to stress due to adrenal stimulation and consequent rise in serum adrenaline and noradrenaline levels^33^. This response also increases during heat stress, especially when animals are raised in the tropics, but also in situations as fear, excitement, and physical exercise^13,32,34^.

SP systems attenuation of heat stress was associated with the regulation of physiologic mediators, such as cortisol. In the present study, evaluation of cortisol and heat-shock protein expression were performed in order to further characterize heat stress in the experimental animals. Heifers and cows grazing the SP system had lower cortisol levels than those in the IR system, probably due to the milder environmental temperatures of this system, corroborating previous researches^9,11^. During summer, heifers and cows in both systems had high cortisol levels, coinciding with higher climatic indexes recorded, in accordance with other trials^16,23^. Additionally, cows in the early postpartum had higher levels of serum cortisol, corroborating previous data, especially if they were primiparous^35^. However, during the autumn, heifers of the silvopastoral system reached basal levels of cortisol (5–10 ng/mL)^36^, while cows maintained cortisol levels above baseline^37^ in both systems. Although a reduction in gene expression of HSP90AA1 in cows allocated in shaded pastures was expected, levels did not differ nor were influenced by variation of climatic conditions, being similar to that found in zebu cattle^38^. It is possible that signaling of thermal stress at the cellular level occurs due to a cumulative exposure, as already reported in some studies^12,16,39^, and it should be considered that these effects may become evident only after longer periods of animal exposure to the stressor stimulus. It is also possible that this variable had not been a specific marker for heat stress in our experimental conditions.

The SP system increased the body weight of heifers and cows. In fact, thermal discomfort compromises food intake and, consequently, can lead to weight and body condition score loss over time^16,40,41^, as observed in the heifers in the unshaded system, mainly because stressed animals have high levels of cortisol^33^. Cows of both systems had lower levels of glucose and higher BHBA, which are indicative of negative energy balance^24^, common in early postpartum close to the peak of lactation. Negative energy balance is more related to dry matter intake than to the amount of milk produced in dairy cows^42^. Even though systems did not impact dry matter intake, in cows of the present study, the average was 1.8% of body weight and the expected for beef cows should be around 2.2%^37^. Therefore, we speculate that the reason for this metabolic condition was the lower dry matter intake observed for these animals independently of systems.

An increase in glucose and reduction in BHBA and NEFA levels observed in February coincided with the improvement of the body condition score of the experimental animals, evidencing the reduction of negative energy balance in this period^37^. Regarding the characteristics of the progeny, male calves had higher birth and weaning weights than female calves, corroborating data previously reported^43^. Previous researches show that the gender of calves can influence 31.4% of weaning weight^43^. Genotype and environment interactions change the phenotypic expression in different environments^44^; however, no statistical difference was observed in weaning weights when calves raised in SP and IR systems were compared. Despite not being a major objective of this work, more specific studies should be carried out to elucidate the effects of SP systems on calves’ performance from birth to weaning.

In general, reproductive performance was not affected by the system. There was no expressive difference in the production of embryo between systems. However, the higher recovery rate of oocytes observed in the SP (74.3 vs 65.3%) might constitute an additional advantage of shaded systems considering that the success in pick-up process is a critical step to in vitro embryo production. However, specific mechanisms of subsequent laboratory steps should be studied to elucidate the equivalent blastocyst rates on D7 from cows raised on shaded or unshaded pastures (42.9 vs 35.9%). This was in contrast to previous results in which an increase in the rate of blastocysts was observed in Nellore heifers raised in the SP system^45^. However, during the summer, lower numbers of total oocytes, viable oocytes, recovery rate, cleaved structures, cleavage rate, and blastocysts on day 7 were observed, evidencing the deleterious effect of warmer seasons on fertility, as previously reported^45,46^.

The increase in BHBA and NEFA observed in May coincides with the decrease in forage quality, indicating a negative energy balance. In this sense, we speculate that higher concentrations of these components could be present in the follicular fluid, impairing postpartum fertility^47^, reducing the rate of maturation, cleavage rate, and in vitro produced blastocysts^48^. However, in that period, the total number of oocytes, viable oocytes, cleaved structures, blastocysts on day 7 and recovery, cleavage, and blastocyst rates were higher than in other months. The most plausible explanation for the absence of heat stress and nutritional effect in embryo production is that the impact could not be immediately observed, possibly the deleterious effects will compromise oogenesis in a long-term moment.

The number of follicles observed was the same in both systems, as expected, since the number of antral follicles in cows is initially influenced by the pre-existing pre-antral population, which is still determined in the embryonic stage. Therefore, a great genetic influence on the number of follicles can be observed in the ovaries of cows^14^. Progesterone concentration was lower at the beginning than at the end of the study, classically characterizing the return to postpartum cyclicity, as previously reported^49,50^.

In a likely scenario of increased demand for food, considering global population growth, the SP system can be used as a sustainable alternative for beef production. Our data confirm previous results about the benefits of SP systems, both in increasing thermal comfort and welfare, as well as in the performance of heifers and cows. Results of this study lead us to ask what would be the minimum period required for female maintenance in the SP systems that would determine higher quality to oocyte and/or embryo.

We conclude that the use of natural shading due to the presence of arboreous components has a decisive influence on the quality of the microclimate created inside the pasture areas, an essential factor in promoting animal comfort. As a consequence, the silvopastoral system was an efficient model to mitigate thermal stress, stimulating the performance of beef heifers and cows. Select physiological parameters and most of the production of embryos indices were only affected by the production system in the warmer months. However, the silvopastoral system increased the recovery rate of oocytes in primiparous cows. Thus, silvopastoral systems can be considered an effective alternative for beef production, as they fulfill the premises of animal comfort and welfare.

## Material and methods

The experimental procedures were performed in accordance with current Brazilian laws and were previously approved by the Ethical Committee on the Use of Experimental Animals of Embrapa Southeast Livestock (CEUA-CPPSE Protocol, Declaration 05/12/2014). The study was carried out and reported in compliance with the ARRIVE guidelines (Animal Research: Reporting of in Vivo Experiments).

### Experiment 1: Thermal responses and performance of beef heifers in intensive rotational or silvopastoral systems

#### Experimental systems and period

The experiment was conducted at Embrapa Southeast Livestock, in São Carlos, Brazil (21° 57′ 42″ S, 47° 50′ 28″ W, 860 m). The climate is tropical, classified as Cwa^51^. Two different grazing systems of *Urochloa brizantha* (cv. Piatã) were used: (1) Intensive rotational system unshaded pastures (IR; 12 ha); and (2) Silvopastoral system: consisting of grazing under *Eucalyptus urograndis* trees (SP; 12 ha), planted in single rows in an east–west orientation, spaced 15 m between rows and 2 m between plants (330 trees/ha). The trial was conducted from December 2015 to May 2016. During the experimental period, the trees had a mean height of 21 m and 20 cm in diameter at a height of 1.3 m from the ground^27^.

#### Microclimatic characterization

Two automatic meteorological stations were used, which were placed in full sunlight in the IR system or placed immediately below the tree stand, in the SP system. Then, for each system, black globe humidity index-BGHI^27^ and heat load index-HLI^22^ were calculated. Currently, many studies have been considering the black globe humidity index as the most accurate indicator of animal comfort than the classical temperature and humidity index-THI^29,52,53^, especially in SP systems^54^. For that, meteorological data were used from 11:00 a.m. to 3:00 p.m., which comprises the period of the day with the greatest thermal challenge^55^.

#### Animals and productive performance

Thirty-two Canchim (3/8 Nellore 5/8 Charolais) beef heifers were used, with 16 months and 249 ± 2 kg at the beginning of the experiment. Previously to the experiment, all heifers were raised on the same IR system, under homogeneous environmental and nutritional conditions. The animals were randomized to both experimental systems (IR, n = 16 or SP, n = 16), preceded by a 15-day adaptation period. Each system consisted of two experimental areas (area repetition) of 6 ha each, in rotational pasture, with six paddocks (0.5 ha/paddock), that were used for 6 days of occupation and 30 days of rest. Adjustments were managed according to the availability of forage, using the “put and take” technique^56^, in order to provide animals with similar forage availability, regardless of the system of production. Pastures were previously fertilized with 202 kg of N per year, divided into five applications. The average stocking rate in both systems was 2.9 ± 0.5 AU/ha. All animals received a mineral supplement and had ad libitum access to water. Daily dry matter intake (DMI; kg DM/day) of the animals was calculated by the following equation:

$${\text{DMI}} = 0.01673 \times {\text{final BW}} + 8.123 \times {\text{ME}} - [\kern-0.15em[ 3.0042 \times {\text{ME}}]^{2} - 3.6262.$$

This considers the final live weight (BW) and the maintenance net energy (ME)^22^. ME was based on the value 0.0717 Mcal/EBW (empty body weight) m^0.75^, as proposed by BRCORTE, 2010^57^. The body weight and body condition score (BCS, 1–9 scale) were evaluated monthly^58^.

#### Evaluation of physiological variables and serum cortisol concentration

The physiological variables related to the animal thermal balance were measured twice a week in the afternoon (2:00 p.m. to 3:30 p.m.) in the following sequence: respiratory rate (breaths/min), heart rate (beats/min), and rectal temperature (°C)^23,32^. Acquisition of images to obtain the body surface temperature was performed twice a week (2:00 p.m. to 3:30 p.m.), in SP and IR systems, with the animals in standing position, in free-grazing condition^30^, individually, with an infra-red 640 × 480 pixels detector equipped with a long-range lens (42° × 32°, 15 mm), thermal sensitivity < 40 mK (< 0.04 °C at 30 °C), temperature range from − 20 to 350 °C, in the manual focus adjustment option. The emissivity adopted was 0.98^59^. The camera was positioned perpendicular to the right antimere of the animal at a distance of approximately 5.0 m^31^. To calculate the back line temperature (°C), individual images of all heifers from each system were used and the mean was determined using a linear tracing of the scapula to the iliac crest^60^. The analysis of the thermograms was done using the IRSoft software, version 4.0 (Testo AG, Lenzkirch, Germany; https://www.testo.com/en/products/thermography-irsoft).

To determine serum cortisol concentrations, blood samples were collected weekly by jugular venipuncture. After clotting, samples were centrifuged at 1350×*g* for 15 min for serum separation. Serum was stored at − 20 °C. Cortisol concentrations were determined by radioimmunoassay (Cortisol Immuchem Coated Tube Kit; MP Biomedicals, LCC Diagnostics Division, USA). The sensitivity and intra-assay coefficient were 0.17 ng/mL and 11%, respectively.

### Experiment 2: Metabolic and reproductive responses of postpartum cows maintained in intensive rotational or silvopastoral systems

#### Experimental system and period

The same experimental systems already described in Experiment 1 were used, with the number of trees per hectare differing to 165/ha, due to the paring of trees. This procedure was done for the maintenance of dynamic equilibrium between pastures cultivation and the exploitation of the arboreal component. The trial was conducted from January to May 2017.

#### Microclimatic characterization

The same methodology already described in Experiment 1 was used.

#### Animals and productive performance

Eighteen primiparous Canchim cows, previously selected with the criterion of the genealogy were used. Females with a high number of ovarian follicles (≥ 14) were allocated to the experimental systems (IR, n = 8 or SP, n = 10) 60 days before the expected day of partum. At the beginning of the experiment, females had a mean age of 41 months, weight of 477 ± 12 kg, and 26 ± 2 days postpartum and remained in a rotational grazing system, as described in Exp. 1. The average stocking rate was 2.3 ± 0.4 AU/ha for both systems. Dry matter intake, body weight, and body condition score were monitored monthly, as described in Experiment 1. At birth and weaning, the calves were weighed, and age at weaning was adjusted to 205 days according to Beef Improvement Federation^61^

#### Evaluation of physiological variables

In addition to back line temperature (as performed in Experiment 1), the trunk temperature was determined by tracing a polygon comprising the ventral and dorsal border of each animal between the scapula and the iliac crest^31^, every 15 days (2:00 p.m. to 3:30 p.m.).

#### Cortisol and heat shock proteins

Blood samples were collected monthly for determining cortisol levels, as described in Experiment 1. The sensitivity and intra-assay coefficient were 0.20 ng/mL and 9%, respectively.

The evaluation of the abundance of HSP90AA1 transcripts in lymphocytes was adopted for the evaluation of heat stress proteins. Blood samples were collected once a month in the morning, 1 day before the ovum pick-up, in lithium heparin-coated evacuated tubes and kept at 24 °C until work up. Peripheral blood mononuclear cells (PBMCs) were isolated from whole blood by density gradient using Ficoll-Paque Plus. The isolated cells were washed three times with PBS and stored at − 80 °C. For total RNA extraction, 1 mL of Trizol (15596026, Thermo Fisher Scientific, São Paulo, Brazil) was used for each sample^62^. The RNA was diluted in 30 μL of nuclease-free water. The concentration and purity of the RNA were verified by spectrophotometer (GE NanoVue Plus, GE, São Paulo, Brazil) by the absorbance at 260 nm and by the 260/280 and 260/230 ratios, respectively. An aliquot of 1 μg of total RNA diluted in 8 μL of the final volume of water was treated with DNAse I to degrade the DNA from possible contamination by genomic DNA during the RNA extraction step. For this, the samples were incubated with DNAse I for 10 min at 37 °C, followed by enzymatic inactivation with EDTA at 65 °C for 10 min. The cDNA synthesis was performed using the High-Capacity cDNA Reverse Transcription kit (Applied Biosystems). The prepared cDNA was stored at − 20 °C until use.

The relative abundance analysis of the HSP90AA1 transcript in mononuclear cells samples was performed by Real-Time PCR. The primers were obtained using the NCBI database (https://www.ncbi.nlm.nih.gov/genbank/). The reactions were performed in a Step One Plus thermal cycler (Applied Biosystems), in 96-well plates and in triplicates. For this, it was used a PowerUp SYBR Green Master Mix probe (Thermo Fisher). The Cycle Threshold (Ct) of each reaction was determined by means of the LinRegPCR software, version 11.0 (http://linregpcr.nl/), identifying the region of greater efficiency of the curve of exponential amplification. Values of Ct for HSP90AA1 were then normalized by Ct values of the reference gene (PPIA)^63^ to obtain the relative abundance values.

#### Metabolites and progesterone assays

Once a month, blood samples were collected by jugular venipuncture for measurement of progesterone (P4), β-hydroxybutyrate (BHBA), non-esterified fatty acids (NEFA), and glucose. After clotting, samples were centrifuged at 1350×*g* for 15 min and serum stored at − 20 °C for further analysis. NEFA and BHBA concentrations were measured by colorimetric enzyme kits (HR Series NEFA-HR(2); Wako Pure Chemical Industries Ltd., Richmond, VA, USA; kit H7587; Pointe Scientific, Inc., Canton, MI, USA). Intra- and inter-assay coefficients were 5.9% and 2.5% for NEFA, and 7.0% and 7.5% for BHBA. Plasma glucose concentration was determined by the quantitative colorimetric method (kit G7521; Pointe Scientific, Inc., Canton, MI, USA). Intra- and inter-assay coefficients were 2.7% and 3.3%, respectively. Progesterone concentrations were determined by a chemiluminescent immunoassay kit (IMMULITE 1000; Siemens Medical Solutions Diagnostics, Los Angeles, CA, USA), and intra-assay coefficient of variation was 4.1%.

#### Ovarian ultrasonography and in vitro embryo production

Once a month, females were submitted to ovum pick-up, on a random day of the estrous cycle, totaling five sessions of aspiration per animal. For ovarian evaluation and ovum pick-up, the animals were submitted to epidural anesthesia with 2% lidocaine. The aspiration system consisted of ultrasound equipment coupled to a multi-frequency microconvex transducer (5.0 MHz), a vacuum pressure pump of 75 mmHg, a rigid hose, and a needle of 0.9 × 50 mm (20G).

All ovarian follicles with a diameter larger than or equal to 5 mm were counted and aspirated. The recovered follicular contents were stored in a 50 mL polypropylene tube containing DPBS, plus 1% fetal bovine serum (F1051, Sigma-Aldrich, Ontario, Canada) and 5 IU sodium heparin/mL, at a temperature between 35 and 37 °C. Cumulus oocytes were counted and evaluated according to morphology (number of cumulus cell layers and cytoplasmic appearance) in grades (GI, GII or GIII), considered as viable oocytes, and also degenerated, without cumulus, and atretic, which were counted to provide the number of total oocytes^64^. The recovery rate was calculated considering the number of total recovered oocytes on the number of observed follicles.

All procedures of in vitro embryo production were done in a commercial laboratory. Briefly, viable oocytes were submitted to in vitro maturation for 24 h, in humidified incubators maintained at 38.5 °C in air with 5% CO_2_. For in vitro fertilization, a previously tested frozen semen from the same batch of a single bull was used. Sperm separation was done by Percoll gradient, and the final concentration was adjusted to 1 × 10^6^ sperm/mL. Oocytes and sperm cells were cocultured for 20 h in the same incubator conditions of in vitro maturation. After in vitro fertilization, zygotes were denuded and submitted to in vitro culture, in the same atmosphere conditions previously described. After 72 h of fertilization, cleaved structures were counted and cleavage rate ([number of cleaved structure/number of viable oocytes × 100] was calculated). Counting of blastocyst stages (initial-Bi, blastocyst-Bl), blastocyst (blastocyst on day 7 number/viable oocyte number) and blastocyst rate (blastocyst on day 7 number/viable oocyte number) was calculated on D7.

### Statistical analysis

Continuous variables were analyzed as repeated measures by the maximum likelihood methodology using PROC MIXED of SAS, version 9.4 (SAS Institute, Cary, USA; https://www.sas.com/). Animal (group) was included in the model as a random effect, and system, month, and interactions as fixed effects. Counting and percentage variables were characterized according to Poisson distribution. These variables were analyzed by a logistic model as repeated measures by PROC GENMOD of SAS, version 9.4 (SAS Institute, Cary, USA; https://www.sas.com/). The black globe humidity index and the heat load index were considered as covariates, and the fixed effects studied in this statistical model were system, month, and interactions between system by month. The level of significance was 5% and the statistical tendency was between 5 and 10%.
